# The Application of a Commercially Available Citrus-Based Extract Mitigates Moderate NaCl-Stress in *Arabidopsis thaliana* Plants

**DOI:** 10.3390/plants9081010

**Published:** 2020-08-10

**Authors:** Johannes Loubser, Paul Hills

**Affiliations:** Institute for Plant Biotechnology, Department of Genetics, Faculty of AgriSciences, Stellenbosch University, Private Bag X1, Matieland 7602, South Africa; phills@sun.ac.za

**Keywords:** anthocyanin, Arabidopsis, BC204, biostimulants, malondialdehyde, proline, salinity, *SOS1*

## Abstract

Aims: The aim of this study was to assess the effect of BC204 as a plant biostimulant on *Arabidopsis thaliana* plants under normal and NaCl-stressed conditions. Methods: For this study, ex vitro and in vitro growth experiments were conducted to assess the effect of both NaCl and BC204 on basic physiological parameters such as biomass, chlorophyll, proline, malondialdehyde, stomatal conductivity, Fv/Fm and the expression of four NaCl-responsive genes. Results: This study provides preliminary evidence that BC204 mitigates salt stress in *Arabidopsis thaliana*. BC204 treatment increased chlorophyll content, fresh and dry weights, whilst reducing proline, anthocyanin and malondialdehyde content in the presence of 10 dS·m^−1^ electroconductivity (EC) salt stress. Stomatal conductivity was also reduced by BC204 and NaCl in source leaves. In addition, BC204 had a significant effect on the expression of salinity-related genes, stimulating the expression of salinity-related genes *RD29A* and *SOS1* independently of NaCl-stress. Conclusions: BC204 stimulated plant growth under normal growth conditions by increasing above-ground shoot tissue and root and shoot growth in vitro. BC204 also increased chlorophyll content while reducing stomatal conductivity. BC204 furthermore mitigated moderate to severe salt stress (10–20 dS·m^−1^) in *A. thaliana*. Under salt stress conditions, BC204 reduced the levels of proline, anthocyanin and malondialdehyde. The exact mechanism by which this occurs is unknown, but the results in this study suggest that BC204 may act as a priming agent, stimulating the expression of genes such as *SOS1* and *RD29A*.

## 1. Introduction

Soil salinity is a global problem, affecting more than 100 countries [1,2] and approximately 20% of the world’s irrigated cropland [3,4]. It is estimated that more than 50% of global arable land will be affected by 2050 [5]. Primary salinisation occurs naturally via precipitation and through groundwater, resulting from rock erosion over long periods of time [6], while secondary or human-induced salinisation is caused by a combination of poor drainage and irrigation [7]. Both combine to change the soil for the worse, leading to the accumulation of ions such as Na^+^, K^+^, Ca^2+^, Mg^2+^ and Cl^−^. Although all of these ions can cause soil salinisation, high concentrations of Na^+^ as a result of excess NaCl is the most prevalent and the subject of most salt-stress related research [8]. This build-up of Na^+^ is the major culprit in the detrimental effects seen on plant growth, but it remains unclear how this is sensed by the plant [9]. 

Saline conditions drastically reduce the yield of major crops, due to the toxic effects of Na^+^ accumulating in plant tissue leading to stunted growth and eventually cell death. High salinity influences all aspects of plant growth and growth stages, leading to ion toxicity, osmotic stress, nutrient deficiency and oxidative stress [10]. Most plant/crop species that are used to feed the global population are highly sensitive to high salinity [11,12].

Salty soil initially induces osmotic stress which results in nutrient imbalances, interruption of membranes and disrupts the plant’s ability to detoxify reactive oxygen species (ROS) which damage the plant at a molecular level [13]. The exact mechanisms by which plants mitigate salt stress have not been fully elucidated, but several role-players have been implicated. 

In response to environmental stimuli such as salt stress, cellular abscisic acid (ABA) levels are increased [11], which triggers the expression of numerous stress-responsive genes [14]. This regulation occurs via transcription factors which are elevated in response to salt stress and also through histone H3 acetylation and methylation which further regulates stress-inducible gene expression [15]. One example is the induction of *RD29A* expression [16]. The increase in ABA levels also stimulates an increase in the production of ROS, which is used in research to monitor intracellular levels of oxidative stress in plants [17,18]. The increase in production of ROS, previously thought to only be a toxic by-product of stress, also serves to provide signaling molecules leading to the production of antioxidants and antioxidative enzymes, forming part of a concerted plant defense reaction [19,20]. If not sufficiently detoxified and scavenged, ROS can cause oxidative damage to proteins, lipids and DNA [21]. The role of ROS in the mitigation of oxidative stress has recently been extensively reviewed [22]. Following prolonged oxidative stress caused by the Na^+^ build-up and subsequent ROS production, ion-toxicity is the inevitable next stage, unless the plant sufficiently deals with the excess Na^+^, which is not the case in most plants. The build-up of Na^+^, which is generally not essential for plants, in the cytosol causes K^+^ deficiency, which disrupts enzymatic processes since K^+^ activates more than 50 key enzymes [9]. 

The accumulation of proline [23], malondialdehyde [24] and anthocyanin [25] are commonly used in research as indicators of salt stress. Proline, a low molecular weight non-enzymatic antioxidant [26], is an osmolyte biosynthesized through the glutamate and ornithine pathways. It plays an important protectory role in plant cells experiencing salt stress [27], alleviating the negative impact of salt by decreasing osmotic stress to maintain membrane integrity and function [26]. Under saline conditions, histone demethylase irreversibly removes the methylation of the *∆^1^-1-pyrroline-5-carboxylate synthetase (P5CS)* coding sequence, leading to the overexpression of *P5CS* and thus an accumulation of proline [28,29]. As an extended effect of the presence of ROS, cell membranes are damaged via the oxidation of acids in the bilayer, a process also known as lipid peroxidation [30]. Lipid peroxidation causes an increase in malondialdehyde levels [2,4], which is the first product formed during free radical-induced damage and the decomposition of polyunsaturated fatty acids in membranes [31]. The production of anthocyanins, also antioxidants, increases in the presence of salt stress as these play a similar protectory role to proline while serving as a signal molecule activating downstream stress-responsive pathways [32,33]. Anthocyanins are also suggested to play roles in quenching ROS, photoprotection and xenohormesis [34]. Elevated levels of anthocyanins in *A. thaliana* have been shown to increase salinity tolerance [35].

Salinity stress induces the expression of a large number of genes and pathways as outlined in two extensive review papers [36,37]. The expression of these genes is often used as indicators of salt stress. The model organism *A. thaliana* has been pivotal in unravelling the Salt Overly Sensitive (SOS) signaling pathway which is involved in salt stress, which was also the first abiotic stress-signaling pathway elucidated in plants [38,39,40]. Independently of ABA signaling, salt stress upregulates the expression of the *AtWRKY8* transcription factor [41] which directly binds to the *RD29A* promoter, leading to upregulation of the gene [37]. The expression of *RD29A* is also induced by cold and drought stress and although its induction and overexpression increase a plant’s resistance to abiotic stress, it was concluded that the RD29A protein is unlikely to serve directly as a protective molecule. The exact function of RD29A is still unknown but it likely serves as a warning signal for abiotic stress responses [42]. NaCl-stress also strongly induces *AtSOT12*, which codes for a sulfotransferase that is also implicated in pathogen resistance via salicylic acid signaling [43].

Three membrane transporters, AtSOS1, AtHKT1 and AtNHX1 are critical Na+ carriers which reduce salt toxicity in plants [12]. AtSOS1 and AtHKT1 are suggested to mediate Na^+^ transport, control ion uptake and spatial distribution of Na^+^ and K^+^ by regulating the expression levels of relevant Na^+^ and K^+^ transporter genes [44]. These membrane transporters remove Na^+^ from the cytoplasm by transporting it into the vacuole or out of the cell [45]. The signaling pathway involves a salt-elicited Ca^2+^ signal in the cytosol, where a myristoylated calcium binding protein, SOS3, activates a serine/threonine protein kinase, SOS2 [46]. The SOS3/SOS2 complex subsequently phosphorylates and activates SOS1 [40,47] which codes for a Na^+^/H^+^ antiporter, expressed and located at the plasma membrane, which exports Na^+^ out of the cytosol into the apoplastic space [45,48,49,50]. *AtSOS1* transcripts can be stabilized by plasma membrane-localized NAPDH oxidase generated ROS [51]. This pathway is highly conserved in plants and its main role seems to be to maintain ion homeostasis [38,52].

Some strategies implemented thus far to address salt stress in agriculture have been to elucidate salt tolerance mechanisms and signaling [11,13,53,54,55,56] and use the information for two genetic approaches to create more salt-tolerant crops. The first approach is creating more salt tolerant varieties through breeding programs, while the second approach is to genetically modify crop plant to be more salt tolerant [4,57,58]. In addition to the usual fertilizers and crop protection agrochemicals used in efforts to improve the plant’s immediate environment, plant biostimulants (PBs) are a novel collection of agrochemicals recently introduced in agriculture. PBs, described as materials that can promote plant growth in minute quantities regardless of nutrient composition [59], have been shown to alleviate the effects of abiotic stress, which includes salt stress, in crop plants. There are a wide variety of commercially available PBs routinely used in agriculture. Examples of commercially patented PBs are extensively reviewed elsewhere [60]. PBs improve plant growth, yield, fruit quality and tolerance to abiotic stress [60,61,62,63,64,65]. Some researchers are developing and finetuning efforts to discover and characterize new PBs suitable for agricultural use [66,67,68]. The priming effect of PBs can be described as preparing the plant for abiotic stress by activating plant defense mechanisms against stress [69,70,71,72].

*Ascophyllum nodosum* extracts (seaweed-extracts), the most well-characterized group of PBs, have been shown to induce salinity tolerance in *A. thaliana* by regulating the expression of stress responsive genes [73] and by modulating miRNAs involved in nutrient acquisition and stress tolerance [74]. There are also other reports where exogenous application of PBs has alleviated or mitigated the effects of NaCl-stress. In sweet pepper, exogenous application of citric acid, humic acid, putrescine and seaweed extracts to unstressed and NaCl-stressed plants increased sugar and potassium (K^+^) content while decreasing Na^+^ and proline content, which was exactly the opposite to the NaCl-stressed plants [75]. In salt -stressed *Solanum lycopersicum* plants, an exopolysaccharide-type PB reduced the levels of proline, Na^+^, phenolic compounds and antioxidant enzyme activity [76]. The effects of PB applications on crop plants to alleviate abiotic stresses such as salinity and improve plant growth have been recently reviewed [61]. BC204 is a citrus-based PB currently used in South Africa, Australia, China and USA as an agrochemical to improve crop growth. There is no published literature which describes the effects of BC204 on plant growth and abiotic stress tolerance. Here, we examined the effects of BC204 on the growth of the model organism *A. thaliana*, and whether BC204 was able to mitigate salinity stress in this species.

## 2. Results

### 2.1. BC204 Increases A. thaliana Above-Ground Fresh and Dry Weight While Increasing Leaf Number under Both Normal and Salinity Stress Conditions

**Figure 1:**
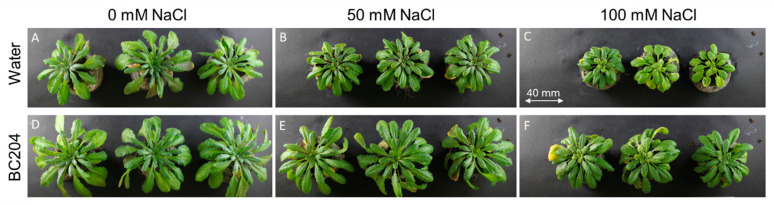
Rosette growth of *Arabidopsis thaliana* plants in response to salt (NaCl) and BC204 treatments. Plants were treated with water (**A**), 50 mM NaCl (**B**), 100 mM NaCl (**C**), 0.01% (*v*/*v*) BC204 (**D**) and combinations of BC204 and 50 mM (**E**) and 100 mM (**F**) NaCl.

**Figure 2:**
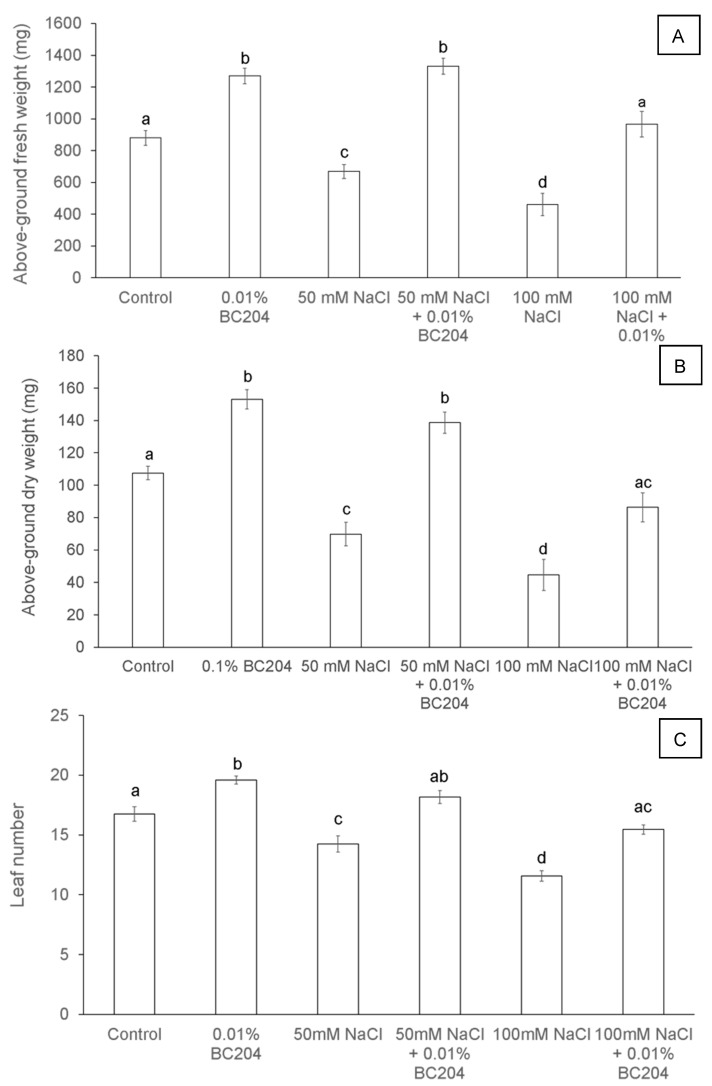
Above-ground fresh (**A**) and dry (**B**) biomass production and total leaf number (**C**) of *Arabidopsis thaliana* plants treated with BC204 and two different concentrations of NaCl. Bars represent the mean of 20 replicates (*n* = 20) ± standard error. Different letters indicate values that were determined by one-way ANOVA with Fisher’s LSD post-hoc test to be significantly different (*p* < 0.05) from the control.

BC204 treatment resulted in enhanced shoot growth of *A. thaliana* Columbia-O plants, even in the presence of 50 mM and 100 mM salinity stress. Plants were germinated and grown on peat discs for three weeks before being treated with 0.01% (*v*/*v*) BC204 every six days and/or NaCl every three days. BC204-treated plants (Figure 1D) were visibly larger and healthier than the untreated control plants (Figure 1A). Salinity stress had a significant impact on plant growth (Figure 1B,C), salt-stressed plants were visibly smaller than their non-stressed counterparts. BC204 treatment resulted in an obvious enhancement of plant growth under saline conditions (Figure 1E,F), such that BC204-treated stressed plants had similar growth characteristics to the untreated unstressed plants. Fresh (Figure 2A) and dry (Figure 2B) mass measurements reflected these observations, with BC204-treated plants being significantly heavier than their control counterparts under both non-stressed and salinity-stressed conditions. BC204 treatment was able to return biomass levels in both 50 mM and 100 mM salt-treated plants to at least those of the untreated control plants. BC204 treatment also increased the leaf number (Figure 2C). The average electroconductivity (EC) of the peat discs for control (water only) and BC204-treated plants were similar, ranging between 0 and 1 dS·m^−1^, while 50 mM NaCl-treated peat discs had an average EC of 10 dS·m^−1^ and 100 mM NaCl-treated disks recorded an average EC of 20 dS·m^−1^.

### 2.2. BC204 Increases A. thaliana Root and Shoot Growth in the Presence and Absence of NaCl-Stress in In Vitro Growth Conditions

**Figure 3:**
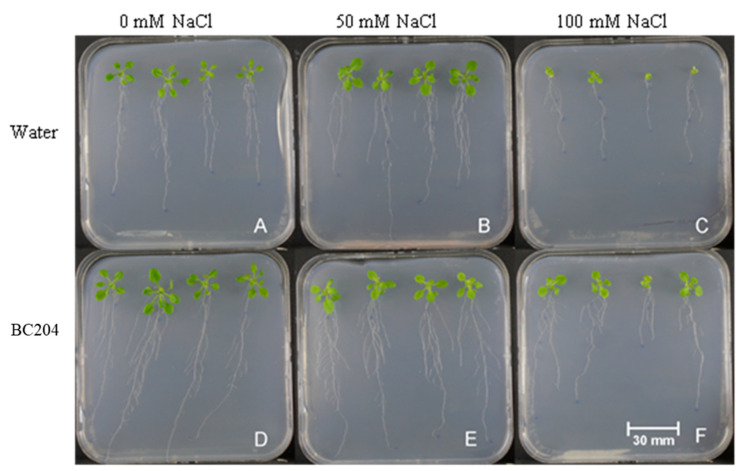
Growth of *Arabidopsis thaliana* seedlings in vitro in response to salt (NaCl) and BC204 treatments. Seedlings were grown on half-strength Murashige and Skoog (MS) media (**A**), supplemented with 50 mM NaCl (**B**), 100 mM NaCl (**C**), 0.001% (*v*/*v*) BC204 (**D**), 50 mM NaCl and BC204 (**E**), and 100 mM NaCl and BC204 (**F**).

**Figure 4:**
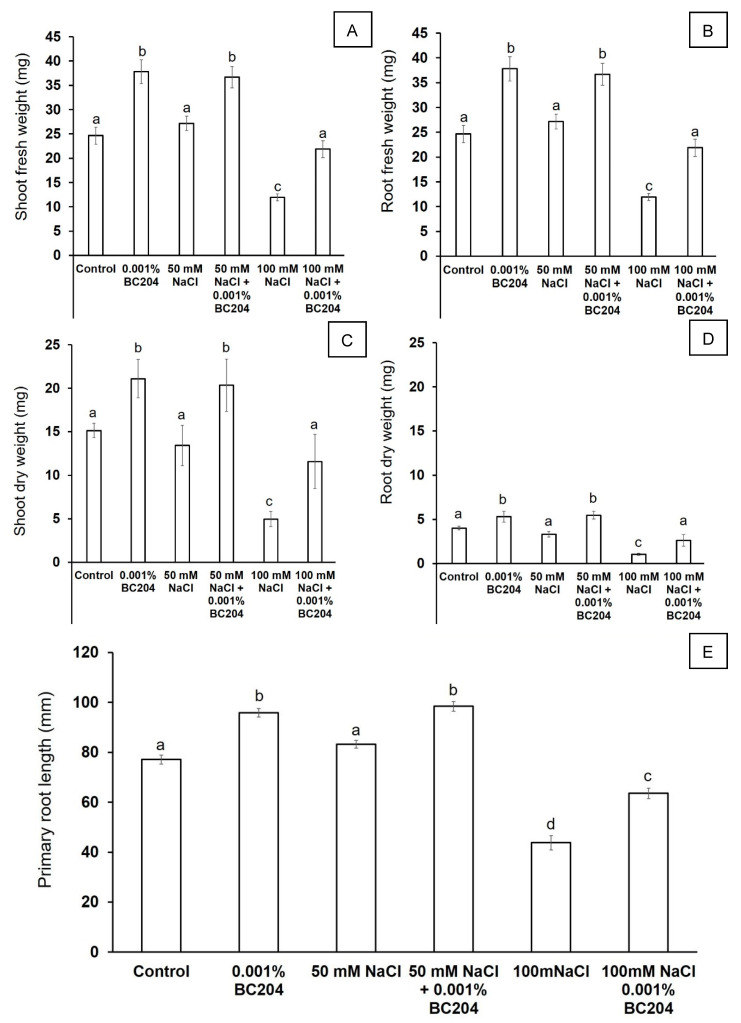
Shoot (**A**) and root (**B**) fresh mass, shoot (**C**) and root (**D**) dry mass and primary root length (**E**) of in vitro grown *Arabidopsis thaliana* seedlings treated with BC204 and NaCl. Bars for tissue fresh weight and primary root length represent the mean of 20 replicates (*n* = 20) ± standard error. Bars for tissue dry weight represent the mean of three pooled samples of 10 replicates (*n* = 3). Different letters indicate values that were determined by one-way ANOVA with Fisher’s LSD post-hoc test to be significantly different (*p* < 0.05) from the control.

To test for the effect of BC204 in vitro, *A. thaliana* seedlings were grown on media supplemented with BC204, NaCl and a combination of both. After germination on media containing no BC204 or NaCl, 4-day old *A. thaliana* Columbia-0 seedlings of identical sizes were transferred to ½ MS media supplemented with either 0.001% (*v*/*v*) BC204, 50 mM NaCl, 50 mM NaCl + 0.001% (*v*/*v*) BC204, 100 mM NaCl or 100 mM NaCl + 0.001% (*v*/*v*) BC204. Roots of BC204-treated seedlings (Figure 3D–F) were visibly larger in comparison to the control (Figure 3A) and NaCl-stressed seedlings (Figure 3B,C). Shoots of BC204-treated seedlings were visibly larger than the untreated control plants on media containing 0 mM and 100 mM NaCl, although no differences were observed between the shoots of 50 mM NaCl treated seedlings and their BC204-treated counterparts. Fresh (Figure 4A,B) and dry masses (Figure 4C,D) of shoots and roots were significantly increased in the presence of 0.001% BC204 after 10 days. BC204 treatment improved biomass accumulation for both shoots and roots under both 50 mM and 100 mM salt to levels comparable to those of the untreated control plants. Primary root length was also increased by BC204, both in the absence and presence of NaCl-stress (Figure 4E).

### 2.3. BC204 Increased Chlorophyll Content, Lowered Stomatal Conductance and Increased Fv/FM in Unstressed and NaCl-Stressed Conditions

**Figure 5:**
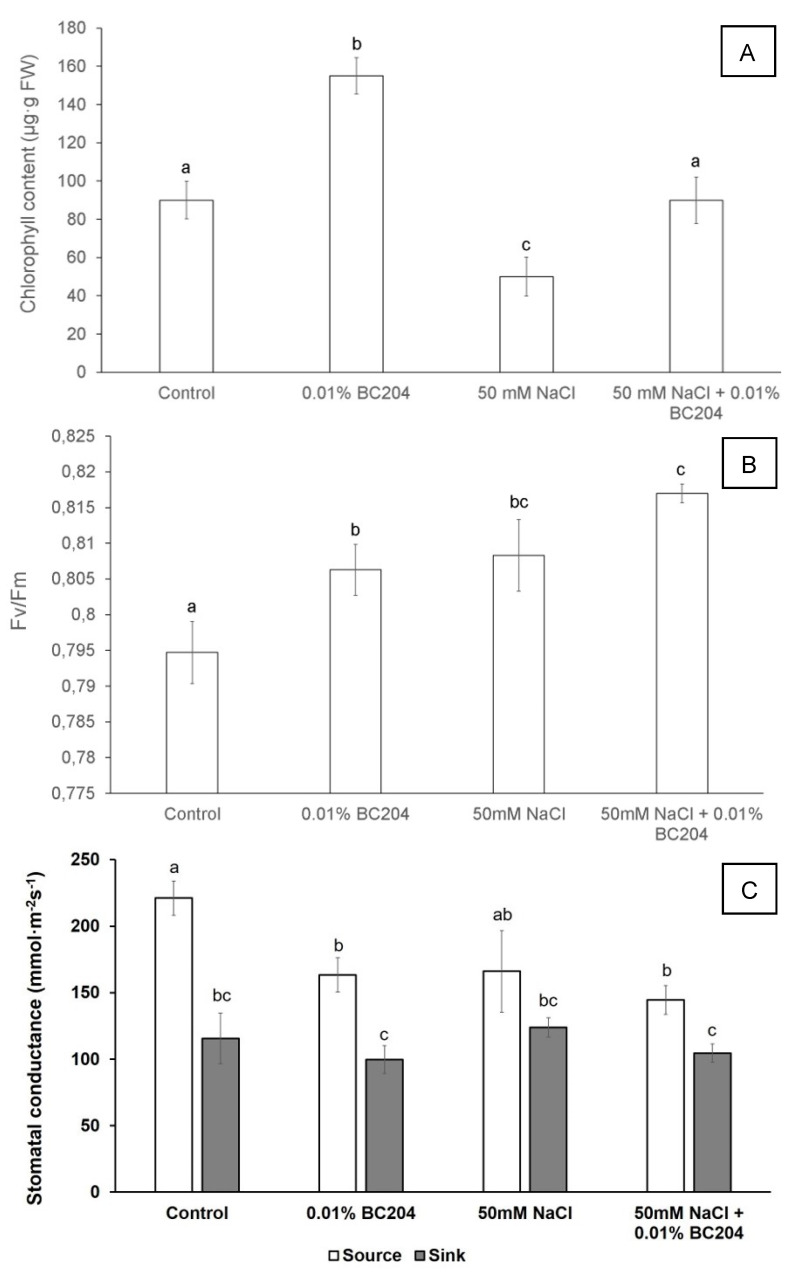
Total chlorophyll content (**A**), Fv/Fm (**B**) and stomatal conductance (**C**) in *Arabidopsis thaliana* plants subjected to NaCl-stress, BC204 treatment and a combination of BC204 and NaCl. Bars represent the mean of 9 replicates (n = 9) ± standard error. Different letters indicate values that were determined by one-way ANOVA with Fisher’s LSD *post-hoc* test to be significantly different (*p* < 0.05) from the control.

To test for the effects of BC204, NaCl, and a combination of both, on Fv/Fm measurements, the same plants from the initial growth experiment were used. The 100 mM NaCl-stressed plants were excluded from these analyses in order to investigate a more moderate salt stress rather than a severe stress [77,78,79]. All measurements were taken and tissues harvested 90 min after the final BC204 treatment. Chlorophyll content increased in BC204-treated plants in comparison to the control. While 50 mM NaCl reduced the amount of chlorophyll in the shoot tissue (Figure 5A), plants treated with BC204 in conjunction with 50 mM NaCl had a chlorophyll content that was similar to that observed in the control group. Fv/Fm values were slightly enhanced by 0.01% BC204 and 50 mM NaCl as individual treatments compared to the untreated control, and a combination of both resulted in the highest Fv/Fm value (Figure 5B). There were no significant differences in stomatal conductance rates in the sink leaves between any of the treatments. However, in the source leaves, 0.01% BC204, 50 mM NaCl and the combination of both all resulted in similarly reduced stomatal conductance levels compared to control plants (Figure 5C).

### 2.4. BC204 Attenuates the High Levels of Anthocyanin, Proline and Malondialdehyde Content Elicited by NaCl-Stress

**Figure 6:**
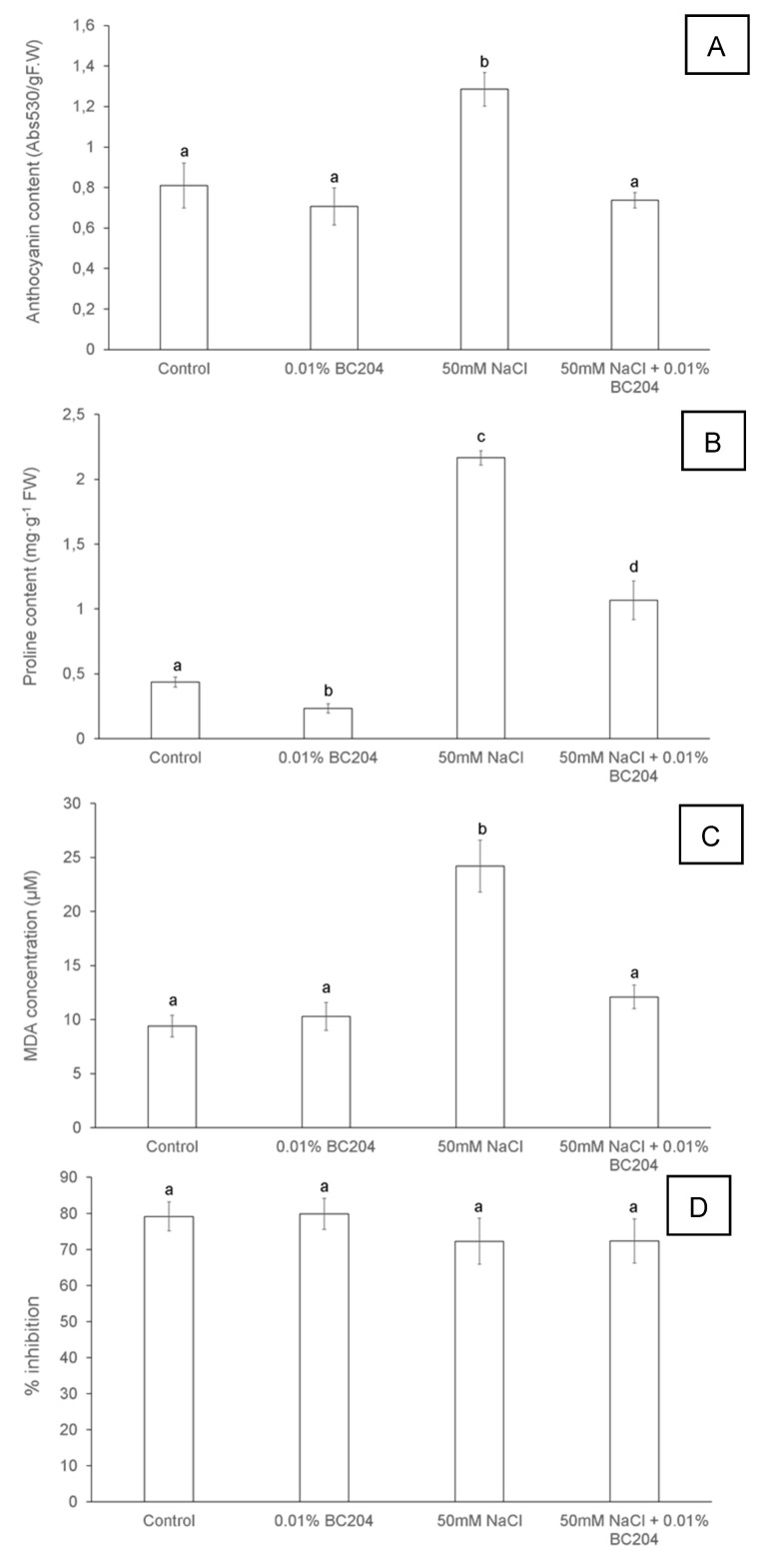
Anthocyanin content (**A**), proline content (**B**), malondialdehyde concentration (**C**) and % SOD inhibition (**D**) in *Arabidopsis thaliana* shoot tissue following BC204 and NaCl treatment. Bars represent the mean of 9 replicates (n = 9) ± standard error. Different letters indicate values that were determined by one-way ANOVA with Fisher’s LSD *post-hoc* test to be significantly different (*p* < 0.05) from the control.

To test for the effect of BC204 and NaCl on anthocyanin, proline and malondialdehyde content, the same plants from the initial growth experiment were used, again excluding the 100 mM NaCl-stressed plants since the aim of the study was to investigate a moderate rather than a severe stress [77,78,79]. Anthocyanin (Figure 6A), proline (Figure 6B) and malondialdehyde (Figure 6C) contents were significantly higher in NaCl-treated plants than in the control plants. BC204 on its own had no direct effect on anthocyanin and malondialdehyde levels, but significantly reduced proline levels even under unstressed conditions. The addition of BC204 to the NaCl-treated plants reduced the levels of anthocyanins and malondialdehyde to those of the control plants. BC204-treatment also significantly reduced proline levels under saline conditions, although these were still elevated compared with control plants. None of the treatments affected SOD activity (Figure 6D).

### 2.5. BC204 Stimulates the Expression of Two NaCl-Responsive Genes

**Figure 7:**
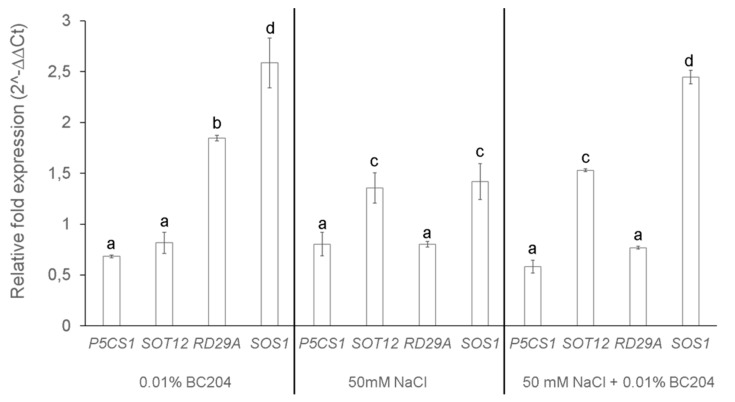
Relative fold expression of NaCl-responsive genes in *A. thaliana* shoot tissue under BC204 and NaCl-stress and a combination of both. Histograms represent relative transcript expression levels of Reverse Transcriptase Quantitative PCR (RT-qPCR) in the shoot tissue of plants treated with BC204 (0.01%), NaCl-stressed (50 mM) and BC204 (0.01%) combined with NaCl-stress (50 mM). Ct values were averaged and normalized to *Eflα* (At1g18070) according to the 2^−ΔΔCt^ method [80]. The expression values for all genes for the untreated control plants were set as 1. Bars represent the mean of three replicates (*n* = 3) ± standard error. Different letters indicate values that were determined by one-way ANOVA with Fisher’s LSD post-hoc test to be significantly different (*p* < 0.05) from the control.

Expression of salt-responsive genes *P5CS1*, *SOT1* and *SOS1* was examined through RT-qPCR to determine the effects of salinity and BC204 treatments. BC204 significantly increased the expression of *RD29A* and *SOS1*, while decreasing the expression of *P5CS1* and *SOT1* (Figure 7). NaCl induced the expression of *SOT1* and *SOS1*, while having no significant effect on the expression of *P5CS1* and *RD29A* (Figure 7). A combination of both BC204 and NaCl resulted in a similar expression profile to NaCl treatment, except for a significant increase in *SOS1* expression levels (Figure 7), similar to that observed following the individual BC204 treatment (Figure 7).

## 3. Discussion

Soil salinity is one of the largest constraints in agriculture, leading to a decrease in plant productivity and agriculture, resulting in approximately 20% of all yield loss [1,2,81]. There is a time constraint on this issue as the world population is growing extensively and a 50% increase in food production will be needed by as early as 2030 [82], while it is estimated that 50% of all arable soil will be salinized by 2050 [36]. However, unravelling these mechanisms and implementing the knowledge gained is a slow process and a large number of questions still remain unanswered [52], a common theme in plant research due to the complexity of the regulatory networks and crosstalk between signaling pathways and plant metabolism as a whole. Our results confirm a mitigating and possible priming effect of BC204 as a PB towards salt-stress, as it enhances tissue fresh and dry mass accumulation, primary root length, and certain stress markers including the accumulation of stress-responsive metabolites and alters the expression patterns of genes involved in NaCl-stress responses.

### 3.1. Bc204 Improved Plant Growth in A. thaliana Grown under Moderate and High Saline Conditions

Plant size, fresh and dry mass and leaf number are the most basic indicators of plant health and are used as initial markers of a positive response to plant growth promotion. The majority of PBs display an increase in these basic indicators of plant growth promotion [83,84,85,86] while plants experiencing abiotic stress are generally smaller with reduced biomass [87,88]. A protein hydrolysate PB from *Medicago sativa* L. has been shown to alleviate salt-stress effects in maize plants by increasing biomass and stimulating plant nitrogen metabolism and antioxidant systems [89]. In tomato plants, *Dunaliella salina* exopolysaccharides alleviated salt stress by increasing tissue fresh and dry weight, root and shoot length while decreasing proline levels, phenolic compounds, Na^+^ levels and antioxidant enzyme activity [76]. In tall fescue turf-grass, foliar application of glycine betaine increased fresh weight clippings under salt stress conditions [90]. Under saline stress conditions, sorghum tissue fresh and dry weight were also increased by humic substances and *Moringa oleifera* leaf extract [91]. In cowpea plants, selenium, glycine betaine and seaweed extract increased biomass under salt stress conditions [92].

In this study, BC204 increased plant biomass under normal growth conditions in both *ex vitro* (Figure 1) and in vitro (Figure 3) experiments. Furthermore, BC204 visibly increased above-ground rosette growth of *A. thaliana* under both 50 Mm and 100 Mm saline conditions (Figure 1), while an increase in root growth was also observed in vitro at the same NaCl concentrations (Figure 3). BC204 significantly increased rosette fresh (Figure 2A) and dry biomass (Figure 2B) of these plants in unstressed and NaCl-stressed conditions, while an increase in leaf number was also observed (Figure 2C). Tissue (root and shoot) fresh and dry biomass and primary root length were also significantly increased in vitro (Figure 3 and Figure 4). The ex vitro experiment simulated salinity conditions which would closely correlate to how NaCl build-up occurs in nature. The addition of 10 mL of either 50 Mm or 100 Mm NaCl to each plant every three days for 21 days resulted in EC values of the peat disks of approximately 10 dS·m^−1^ and 20 dS·m^−1^ respectively at the end of the experiment. Moderately saline water (primary drainage water and groundwater) has an EC of 2 to 10 dS·m^−1^ while highly saline water (secondary drainage water and groundwater) falls between 10 and 25 dS·m^−1^. Irrigation water usually has a measurement of 0.7 to 2 dS·m^−1^ [79]. Most studies on salt responses focus more on short term responses (6 to 48 h) and single salt treatments, which could often simulate salt shock rather than salt stress [93]. Also, most studies focus on the response in the roots rather than the shoot tissue, since roots represent the site where the plants directly experience the stress [94,95].

Studies investigating PB action in vitro are rather scarce, with only a few reporting that certain PBs improve root growth of plants in vitro [96] or an increase in biomass production under saline conditions [97]. For the in vitro experiment in this study, 100 Mm NaCl would be regarded as high salt stress, but *A. thaliana* can grow and survive in concentrations of up to 150 Mm NaCl, which is regarded as salt shock [93,98]. *A. thaliana* grows relatively normally in 50 Mm NaCl [77], as shown in our results (Figure 3). BC204 stimulated root growth under saline conditions (Figure 4). A reduction in primary root length is one of the most common indicators of salt stress in plants [99,100]. Primary root length was significantly reduced in vitro on media containing 100 mM NaCl, however, a significant increase in primary root length was observed in these plants where BC204 was added to the medium in addition to the salt (Figure 4E). To narrow the scope of the study, further analysis was conducted only on peat-grown plants treated with 50 mM NaCl, in order to investigate plant response to moderate salinity stress (~10 dS·m^−1^).

### 3.2. BC204 Increased Total Chlorophyll Content while Reducing Stomatal Conductivity in Unstressed and NaCl-Stress Conditions

It is well documented that abiotic stresses such as salinity stress inhibit photosynthesis, including via stomatal closure. The exact mechanism by which salinity affects photosynthesis, however, remains largely unclear [87]. Since photosynthesis is the main driver of energy production and storage in plants, it is tightly regulated and highly sensitive to environmental stimuli [101]. Chlorophyll, a photosynthetic pigment, is one of the most important components of photosynthesis [102]. An increase in chlorophyll content usually correlates with an increase in photosynthesis and subsequent increase in plant biomass [103]. It is well-documented that under saline stress conditions, chlorophyll content decreases [64,104,105,106,107]. In several reports, PB treatment led to a significant increase in total chlorophyll [108,109,110]. PBs have also been shown to preserve chlorophyll in plants under abiotic stress conditions [67,111]. In this study, BC204 mitigated the reduced total chlorophyll levels observed in 50 mM NaCl-stressed plants, returning these to the same levels as in the untreated control plants (Figure 5A). A significant increase in chlorophyll was also recorded in unstressed plants treated with BC204 (Figure 5A), which may partly explain the enhanced biomass accumulation observed in these plants via its contribution to possible enhanced photosynthesis.

Chlorophyll fluorescence can also be an indicator of the level of stress the plant experiences. Salt stress has been reported to decrease Fv/Fm values [112]. Slight decreases in Fv/Fm have also been reported in *A. thaliana* under salinity stress [113]. In another study, exposure to either 100 Mm or 150 Mm NaCl resulted in Fv/Fm values of approximately 0.78 and 0.75 respectively, compared to the control of 0.8 [114]. Atonik, a PB, did not affect Fv/Fm measurements [115]. BC204, 50 Mm NaCl and a combination of both significantly increased Fv/Fm values compared to the control (Figure 5B).

Plants regulate stomatal conductance to optimize carbon uptake with respect to water [116]. Stomatal conductance is also used in plant research as an indicator of the level of stress the plant experiences [13] and is mediated through ABA signaling [54]. Increased stomatal conductivity is therefore an indication that plants are experiencing no to low levels of environmental stress. Under saline conditions, plants experience a variety of stresses including water stress. In order to prevent water loss, plants close their stomata which lead to a decrease in carbon dioxide in chloroplasts [93]. Under saline conditions, foliar application of glycine betaine as a PB significantly increased stomatal conductance of tomato leaves [117]. In the source leaves in *Arabidopsis*, BC204, 50 Mm NaCl, and a combination of both all reduced stomatal conductance compared to the control (Figure 5C). This was a surprising result as PBs have been reported to increase stomatal conductivity [115,118,119]. Plants close their stomata when they experience abiotic stress in order to prevent water-loss [120]. The lowered stomatal conductance could have been a short-term priming response to the BC204 treatments, as only one measurement was taken at the end of the experiment, 90 min after the final BC204 treatment. Alternatively, increased chlorophyll levels and enhanced Fv/Fm by BC204 allowed the plants to photosynthesize more efficiently without the need to open their stomata further. This remains highly speculative since Fv/Fm as an indicator of stress was measured, and photosynthesis was not measured directly. The lack of effect of any of the treatments on stomatal conductance in the sink leaves is most likely explained by the fact that these leaves are still growing and developing and are not yet fully photosynthetic.

### 3.3. BC204 Attenuated Increased Anthocyanin, Proline and Malondialdehyde Levels under NaCl-Stress

Anthocyanin and proline accumulation under stress-conditions, such as NaCl-stress, provides important protection in plant leaves [31] possibly mediated by salicylic acid [121]. BC204 reduced the levels of both anthocyanin (Figure 6A) and proline (Figure 6B) in shoot tissues of NaCl-stressed plants. Proline is involved in membrane stability while malondialdehyde is a breakdown product of unsaturated fatty acids [122]. Flavonoids, one of the major ingredients in BC204, are known to be synthesized under stress conditions in the plant. If BC204 provides the plants with anthocyanin-like metabolites or precursors, the plant does not have to produce these itself and can rather invest its energy into primary metabolism such as biomass production. This, however, remains highly speculative as the concentration of BC204 used in the treatments is extremely low and would be unlikely to induce such a major metabolic process. Oxidative stress is known to increase the activity of antioxidant enzymes such as ascorbate peroxidase, phenol peroxidase, glutathione peroxidase, catalase and dismutase [17,18]. The percentage (%) SOD activity, however, remained unchanged under all experimental conditions (Figure 6D).

### 3.4. BC204 Treatment Alters the Expression of Genes Involved in the Salt Response

NaCl-stress induces the expression of a large number of genes, some of which have been flagged as markers for NaCl-stress [123]. For example, NaCl-stress commonly induces the expression of *RESPONSIVE TO DESICCATION* (*RD29A*) [59], *SALT OVERLY SENSITIVE* (*SOS1*) [124] and *SULFOTRANSFERASE 12* (*SOT12*) [43]. *SOT12* codes for a sulfotransferase known to sulfonate salicylic acid [43]. The up-regulation of *SOT12* by BC204 suggests a possible activation of defense against biotic stress as well as abiotic stress, something not investigated in this study.

The expression of *RD29A* is induced by abiotic stresses such as cold temperatures, drought and saline conditions [124,125], while ABA has also been also been shown to up-regulate the expression of *RD29A* [126]. PBs developed from brown seaweed extracts have been shown to increase the expression of *RD29A* in *A. thaliana* [127], acting as a priming agent. *RD29A* and its homologue, *RD29B*, code for enzymes unlikely to be directly involved in a protectory role in abiotic stress although their exact functions are still unknown [42]. Transcript abundance of *RD29A* is the highest approximately 2 h after salt treatment [16]. In our results, NaCl treatment did not induce the expression of *RD29A* (Figure 7). This is strange but could be explained by the fact that plant tissue was harvested at the end of the experiment, meaning that possible elevated levels of *RD29A* returned to normal at the time of harvesting.

In the absence of *SOS1* expression, plants are highly susceptible to salt stress [128] while the overexpression of this gene results in an increase in salt tolerance [58]. SOS1 is suggested to also be involved in the control of long-distance Na^+^ transport from the root to the shoot, where under mild salt stress it loads Na^+^ into the xylem and under severe salt stress it retrieves Na^+^ from the xylem [50]. Although the expression of *SOS1* is generally more abundant in root tissue under saline conditions, NaCl has also been shown to up-regulate its expression in shoot tissue [124]. As *SOS1* mRNA has a half-life of approximately 10 min [51], expression might have been even higher if the plant tissue was harvested earlier than 90 min after treatment.

*P5CS1* codes for an enzyme involved in a rate-limiting step of proline biosynthesis [129]. The unchanged levels of expression of *P5CS1* in the presence of BC204 is in contrast to the reduction in proline content observed in this study following BC204 treatments, and particularly with the increase in proline levels observed following 50 mM salt treatment. As mentioned, the expression of this gene is a rate-limiting step in proline biosynthesis and is also subjected to feedback mechanisms. High levels of proline have been shown to suppress the expression of proline biosynthesis genes in *A. thaliana* [130] and *Enterobacteriaceae* [131]. It is possible that at the time of tissue harvesting, the *P5CS1* mRNA levels had been reduced in response to feedback inhibition, although the metabolite levels remained elevated in response to the stress.

BC204 could aid the plant in dealing with salt stress by stimulating the expression of *RD29A* and *SOS1* in the absence of salt stress. BC204 therefore possibly primes the plant by activating the SOS pathway, which is known to improve salt stress tolerance in plants [38]. Although the function of RD29A is still unknown [42], a study revealed that *RD29A* promoter functions in almost all tissues and organs in vegetative plants during water deficiency [132]. Therefore, it is hypothesized that *RD29A*-induced expression by BC204 further serves as possible priming.

## 4. Materials and Methods

### 4.1. Plant Material and Growth Conditions

For pot trials, *A. thaliana* (ecotype Columbia-0) seeds were surface decontaminated via vapour sterilization from an adapted protocol [133] by placing open microcentrifuge tubes containing the seed under a glass dome with a beaker containing 100 mL sodium hypochlorite and 2 mL hydrochloric acid (37%) for 4 h. After vapor sterilization, seeds were sown onto peat disks (Jiffy^TM^ no. 9, Johannesburg, South Africa), subjected to seed stratification (48 h, 4 °C) before being placed in controlled conditions (10 h light, 22 ± 1 °C, 14 h dark, 18 ± 1 °C, 75% relative humidity). As the seeds germinated, excess seedlings were removed with forceps until only one seedling remained on each peat disc. Care was taken to ensure that all remaining seedlings were of the same size. Plants were maintained for three weeks and received no fertilizer. Plants were arranged randomly within the growth chamber and harvested in a random order. Plants were treated with 10 mL 0.01% BC204 weekly, starting 21 days after germination (DAG). Also starting 21 DAG, NaCl-treated plants were treated with 10 mL NaCl solution (either 50 mM or 100 mM) every three days via a soil drench. BC204 and NaCl treatment continued for 3 weeks and all tissues were harvested 3 days after the final treatment. Although *A. thaliana* can tolerate and grow normally at 50 mM NaCl [78], the salt concentration in the peat discs was gradually increased to simulate a moderate salt stress rather than imposing an immediate salt shock [78]. Control plants were watered with 10 mL dH_2_O and all plants were similarly watered on non-treatment days to prevent the peat discs from drying out. Electroconductivity was measured by at room temperature by combining the wet peat from three plants and gently compressing it before inserting the electrodes from a Procheck soil conductivity sensor (Decagon Devices, Pullman, WA, USA). This was repeated three times for each treatment group.

For in vitro trials, *A. thaliana* seeds were surface decontaminated via vapor sterilization as described above. Surface decontaminated seed was sown onto petri dishes containing half-strength Murashige and Skoog (MS) media (Sigma-Aldrich, St Loius, MO, USA) solidified with 0.9% (*w*/*v*) Phytoagar (DUCHEFA Biochemie, Haarlem, The Netherlands) with the pH adjusted to 5.8 using potassium hydroxide (KOH). Growth media were sterilized by autoclaving for 20 min at a temperature of 121 °C and pressure of 103 kPa. Five DAG, seedlings were transferred to a CELLSTAR^®^ petri dish (120 mm × 120 mm) (Greiner Bio-One, Frickenhausen, Germany) with media supplemented with 50 mM NaCl, 100 mM NaCl, 0.001% BC204 and combinations of these. The plates were placed almost vertically under cool white fluorescent tubes (Osram L 58V/740, 50 μmol photons.m^−2^·s^−1^) in controlled conditions (16 h light, 23 ± 2 °C, 8 h dark, 18 ± 1 °C, 75% relative humidity). After 10 d further growth, the plates were opened and photographed with a Canon camera (model E0S 550D). Primary root length was determined using ImageJ (version 1.49) software [134].

### 4.2. Stomatal Conductance, Fv/Fm Measurements

Ninety minutes after the final BC204 treatment, 12 plants from each treatment group (Control, 0.01% [*v*/*v*] BC204, 50 mM NaCl, 50 mM NaCl+ 0.01% [*v*/*v*] BC204) were selected for measurement of stomatal conductance, using a SC-1 leaf porometer (ICT International, Armidale, Australia), and Fv/Fm, using an 0S30p+ chlorophyll fluorometer (Optiscience, Hudson, NH, USA).

### 4.3. Total Chlorophyll Extraction and Quantification

Chlorophyll was extracted from plants grown on peat disks using an adapted protocol [135]. Nine plants were ground to a fine powder using liquid nitrogen in a pre-chilled mortar and pestle. Thereafter, 1 mL dimethylsulfoxide (DMSO) was added to approximately 50 mg ground tissue in a microcentrifuge tube. The extract was vortexed and centrifuged at 10,000× *g* for 2 min. The supernatant was removed to a fresh tube and retained. The pellet was re-extracted twice in further 1 mL aliquots of DMSO and the supernatant liquids combined in a microcentrifuge tube. Absorbance of the samples was measured in triplicate at 645 nm and 663 nm against a DMSO blank. Total chlorophyll was calculated using the formula: (0.0202 × A_663_) + (0.00802 × A_645_).

### 4.4. Anthocyanin, Proline, Malondialdehyde and Superoxide Dismutase (SOD)

Anthocyanins were extracted from plants grown on Jiffy peat disks (Jiffy^TM^ no.9, South Africa) using an adapted protocol [136]. Nine plants were randomly selected per treatment, flash frozen in liquid nitrogen and ground to a fine powder in a pre-chilled mortar and pestle. Total anthocyanins were extracted using a methanol/acetic acid/H_2_O (9:1:10) buffer. Absorbance was measured at 530 nm and 637 nm, then anthocyanin content was calculated as A_530_ − (0.25 − A_637_) and expressed as Abs530/g·FW.

Proline content was determined using an adapted protocol [137]. Nine plants were selected at random, pooled, flash frozen and ground in liquid nitrogen using a mortar and pestle and proline extracted using approximately 50 mg tissue and 5 µL/mg FW 3% (*w*/*v*) sulfosalicylic acid (SAS). Samples were vortexed and centrifuged at 10,000× *g* for 5 min. A 100 µL aliquot of the supernatant was added to 500 µL of a reaction mixture of 3% (*w*/*v*) SAS/glacial acetic acid/acidic ninhydrin (1:2:2). This mixture was heated at 95 °C for 60 min before being centrifuged at 10,000× *g* for 5 min. Absorbance was measured at 520 nm.

Malondialdehyde was extracted and quantified according to Rao and Sresty [136]. Leaf tissue was homogenized with 0.1% (*w*/*v*) trichloroacetic acid (TCA), centrifuged at 10,000× *g* for 5 min and the supernatant mixed with 20% (*w*/*v*) TCA and 0.5% (*w*/*v*) thiobarbituric acid (TBA). The mixture was boiled for 15 min before being transferred to ice and centrifuged at 10,000× *g* for 2 min. The absorbance of the supernatant was measured at 532 nm. SOD activity was determined according to manufacturer’s protocol using the Invitrogen^TM^ K335-100 Superoxide Dismutase (SOD) Activity Colorimetric Assay kit (ThermoFisher Scientific, Waltham, MA, USA).

### 4.5. RNA Extractions and Reverse Transcriptase Quantitative PCR (RT-qPCR) Analysis

Nine plants from each treatment were randomly selected and pooled into three representative samples (3 plants per sample), flash frozen, ground in liquid nitrogen using a mortar and pestle and total RNA extracted in a Maxwell^®^ 16 AS2000 Instrument with the Maxwell^®^ 16 Total RNA Purification Kit, as per the manufacturer’s protocol (Promega, Madison, WI, USA). One microgram of total RNA was used to obtain complementary DNA (cDNA) via reverse transcription using an oligo(dT)18 primer and RevertAid reverse transcriptase (Thermo Scientific^TM^, Waltham, MA, USA), according to manufacturer’s protocol. The PowerUp^TM^ SYBR^TM^ Green Master Mix kit and the QuantStudio 3 Real-Time PCR System was used for reverse transcriptase quantitative PCR (RT-qPCR) analysis and the relative expression calculated using the 2^−ΔΔCT^ method [80]. Expression of each gene for the untreated control plants was set as 1. *Ef1α* (At1g18070) was used for as an internal control as it has previously been shown to be a suitable reference gene for *A. thaliana* under abiotic stress conditions, including salt stress [138]. Primer sequences for RT-qPCR analyses are given in Supplementary Table S1.

### 4.6. Data and Statistical Analysis

All experiments were independently replicated at least three times to ensure reproducibility. Statistical significance between control, treated and NaCl-stressed groups was determined by the one-way ANOVA function in Excel followed by the Fischer’s least significant difference (LSD) test at the 0.05 probability level.

## 5. Conclusions

BC204 stimulates plant growth under normal growth conditions by increasing above-ground shoot tissue and root and shoot growth in vitro. BC204 also increases chlorophyll content while reducing stomatal conductivity. BC204 furthermore mitigates moderate salt stress (10–20 dS·m^−1^) in *A. thaliana*. Under salt stress conditions, BC204 reduces the levels of proline, anthocyanin and malondialdehyde. The exact mechanism is unknown, but the results in this study indicate that BC204 acts as a priming agent in some sense, stimulating the expression of genes such as *SOS1* and *RD29A*.

This study focused on salt stress at a single growth stage, at the end of vegetative growth. However, investigating how the plant reacts to BC204 during oxidative stress/early salt stress will be valuable as it will give insight into a possible priming effect of BC204 during earlier stages of plant growth. Ion measurements (Na^+^ and K^+^) will also be valuable to shed light on the effect of BC204. For gene expression, several samples should be harvested at different time points within the experiment to account for the circadian control of specifically genes like *RD29A* [16].

There is an overlap in how plants perceive and respond to salt, drought and, to a lesser extent cold stress as many genes that are regulated by salt stress also respond to cold or drought stress [39]. Therefore, it is possible that BC204 could have similar alleviating effects on these other abiotic stress conditions. This, however, remains purely speculative and an interesting topic for future investigation.

## Supplementary Materials

The following are available online at https://www.mdpi.com/2223-7747/9/8/1010/s1, Table S1: Primer pairs sequences for RT-qPCR analysis.

## Abbreviations

ABA	abscisic acid
DAG	days after germination
EC	electroconductivity
g·FW	gram fresh weight
LSD	least significant difference
MS	Murashige and Skoog
NaCl	sodium chloride
P5CS	Δ^1^-1-pyrroline-5-carboxylate synthetase
PB	plant biostimulant
PCR	polymerase chain reaction
RD29A	responsive to desiccation
ROS	reactive oxygen species
RT-qPCR	reverse transcriptase quantitative PCR
SOD	superoxide dismutase
SOS	Salt Overly Sensitive
SOT12	sulfotransferase 12
