# Supplementary material for: The Application of a Commercially Available Citrus-Based Extract Mitigates Moderate NaCl-Stress in Arabidopsis thaliana Plants
Source: Plants (Basel). 2020 Aug 10;9(8):1010. doi: 10.3390/plants9081010 (PMC7465524; doi:10.3390/plants9081010)
Supplement: Supplementary file 1 [file plants-09-01010-s001.pdf]

Supplementary material

Table S1. Primer pairs sequences for RT-qPCR analysis.

Gene ID	Gene name	Forward primer (5′-3′)	Reverse primer (5′-3′)	Amplicon size	Annealing temperature
At2g39800	*P5CS1*	AGGTCATGCTG ATGGAATCTGT	GGCTGCTGGAT AGTCCAACTT	103 bp	60°C
At5g52319	*RD29A*	GAGCTCCGTTG GGAGGAAAT	GGTTCTCCGTC AAATCCCGT	103 bp	60°C
At2g03760	*SOT12*	CGAAAAAGCG GTTGAAGCGT	GATTCTCGCGG CTTGCATAC	95 bp	60°C
At2g01980	*SOS1*	CCCAGCTCAA GGTCTCGTTT	TTCAGAGGAA GCTGACACGC	96 bp	60°C
At1g18070	*EF1α*	CGAAAACCCT AGACACCTCGT	TCTGAAAGGA GTCTTGCGGC	105 bp	60°C
